# Choosing PD-1 Inhibitors in Oncology Setting, Left or Right?—Lessons From Value Assessment With ASCO-VF and ESMO-MCBS

**DOI:** 10.3389/fphar.2020.574511

**Published:** 2020-12-18

**Authors:** Qian Jiang, Mei Feng, Youping Li, Jinyi Lang, Hua Wei, Ting Yu

**Affiliations:** ^1^ Sichuan Cancer Hospital and Institute, Sichuan Cancer Centre, School of Medicine, University of Electronic Science and Technology of China, Chengdu, China; ^2^ China Chinese Evidence-Based Medicine Centre, West China Hospital, Sichuan University, Chengdu, China; ^3^ School of Pharmacy, Chengdu Medical College, Chengdu, China; ^4^ Department of Pharmacy, Dujiangyan People’s Hospital, Dujiangyan Medical Center, Dujiangyan, China

**Keywords:** nivolumab, pembrolizumab, value, framework, pharmacoecnomics

## Abstract

**Background:** Influx of innovative therapies and dramatic rise in prices have been prompting value-driven decision-making. Both the American Society of Clinical Oncology (ASCO) and the European Society for Medical Oncology (ESMO) have independently proposed value assessment frameworks.

**Objectives:** To comprehensively examine the value of nivolumab and pembrolizumab by two value assessment frameworks with a cohort of published randomized controlled trials and offer insight into the association between these two frameworks.

**Methods:** Trials were identified with a cutoff date of Nov 30th, 2019. Receiver operating characteristic curves were generated to establish the predictive value of ASCO-VF score to meet ESMO-MCBS grade and discriminate the agreement of these two value assessment tools. Spearman correlation was used to assess the association between monthly cost and ASCO-VF score/ESMO-MCBS grade.

**Results:** 19 randomized controlled trials were eligible. seven (36.8%) trials were of treatment included nivolumab while 12 (63.2%) pembrolizumab. 8 (42.1%) of the trials were of treatments for non-small-cell lung cancer, 5 (26.3%) for melanoma, 2 (10.5%) were for head and neck squamous cell carcinoma, 2 (10.5%) for gastric or gastro-oesophageal junction cancer and 1 (5.3%) for urothelial cancer and renal-cell carcinoma respectively. ASCO scores ranged from 7 to 94.7 with median 40.90. 11 (57.9%) trials met the ESMO criteria for meaningful value achieved. Of 14 trials not meeting the ASCO cutoff score, only 8 did not meet the meaningful ESMO criteria. Agreement between these two frameworks thresholds was only fair (*κ* = 0.412, *P*＜0.05). A negative correlation was noted between increment monthly cost and value assessment results.

**Conclusion:** There is only fair correlation between ASCO and ESMO value assessment frameworks. Not all treatment with nivolumab and pembrolizumab meet valuable thresholds.

## Introduction

Influx of innovative therapies, particularly the targeted drugs and immunotherapies have marked major therapeutic advances in oncology. Nevertheless, dramatic rise in prices of these drugs supports the growing concern whether their value demonstrated by evidence is commensurate with the high prices and is challenging to put into practice (Promoting Value, 2018). Favorable evidence for high-value drugs could incentivize the development of novel drug regimens and facilitate conversations in clinical practice. To facilitate value-driven decision-making, an evolving field from the perspective of stakeholders—physicians, patients, health care insurers, etc*.*, including the American Society of Clinical Oncology (ASCO) (Schnipper et al., 2015; Schnipper et al., 2016) and the European Society for Medical Oncology (ESMO) (Cherny et al., 2015; Cherny et al., 2017) have independently proposed frameworks as unbiased tools for systematic assessment of value of anticancer drugs, justifiably evaluating high quality therapies affordable for various cancer disease states.

To date, only a few reports have focused on application of two prominent tools-ASCO Value Framework (ASCO-VF), or the ESMO Magnitude of Clinical Benefit Scale (ESMO-MCBS), or both according to a contemporary cohort of randomized controlled trials (RCTs) to assess the value of anticancer drugs, suggesting that not all approved drugs were significantly associated with meaningful value and additionally exploring the extent of concordance or discordance between these two respective frameworks (Del Paggio et al., 2017; Foote et al., 2017; Vivot et al., 2017; Djatche et al., 2018). However, studies of value assessment were limited.

New anticancer drugs approved by the Food and Drug Administration are expected to be of high value. For example, programmed cell death (PD-1) inhibitors (nivolumab and pembrolizumab) have revolutionized cancer therapy and have shown potential efficacy for a wide range of tumor types based on data from published studies. These approvals have resulted in a widespread prescribing of PD-1 inhibitors in real-world clinical practice. Up to now, numerous RCTs have reported the benefits and safety of PD‐1 inhibitors. Unfortunately, not all cancer patients—now and in the future—might be able to afford these drugs because of their high prices. Furthermore, patients know the high prices of these drugs but not their value, or misunderstand the drugs prices and their value, both of which might stifle innovation in the development of anticancer drugs and in turn prevent the patients from achieving optimal cancer care.

Overall, we performed this study to comprehensively integrate the value of two PD-1 inhibitors (nivolumab and pembrolizumab) by ASCO-VF and ESMO-MCBS in a cohort of RCTs and offer insight into the association between these two frameworks as an important structured evidence for clinicians in making clinical decisions.

## Methods

### Study Cohort and Eligibility Criteria

Phase III RCTs that compared nivolumab and pembrolizumab alone or in combination with chemotherapy, hormonal therapy, other targeted agents, etc*.*, to the same regimen without them used in the intervention group irrespective of the cancer type and stage were identified.

Reports of secondary, subset, or pooled data, phase I or II trials, animal studies, or trials that assessed drug delivery or single-drug dosing schedules were excluded.

### Literature Search

Systematic search of electronic databases including the Cochrane Controlled Trials Register on the Cochrane Library, MEDLINE, EMBASE, and Science Citation Index was conducted using the terms *nivolumab, pembrolizumab and PD-1 inhibitor*, with a cutoff date of Nov 30, 2019. Both MeSH and free text terms were used to identify relevant articles. Reference lists of pertinent retrieved articles were reviewed for additional studies, and ClinicalTrials.gov was also checked in June 2019 to ensure that data from previously published trials were updated on the registry.

### Study Process

Two authors (HW and HL) independently conducted the literature search, screened titles and abstracts for potential eligibility and full texts for final eligibility. In case of disagreement, a consensus was reached through discussion.

Treatments were classified as curative or palliative according to the trial population. Two reviewers extracted the data using a standardized extraction form, including but not limited to *the trial name, phase, cancer type, PD-1 inhibitor used, dosing schedule, follow-up time and outcomes* in accordance with the ASCO-VF and ESMO-MCBS for all eligible studies. ASCO-VF scores and ESMO-MCBS grades were independently recorded. Any discrepancies were discussed among all authors to establish a final score or grade.

To assess the monthly cost of therapeutic regimen including the cost of all anticancer drugs in the study regimen, we used the price for branded and generic drugs recorded in the Hospital Information System (HIS). Monthly costs were calculated over an average of 30 days based on the dosage schedule in all eligible trials for a patient weighing 60 kg with a body surface area of 1.70 m^2^. Ultimately, incremental monthly drug costs as the difference between the experimental and control groups were reported. The most expensive one was recorded if there were several options of therapeutic regimen in the control group. All therapeutic regimens were adjusted to provide the price per 4-week period.

### Statistical Analysis

All data were collected using structured Excel sheets designed for this study. Statistical analysis was performed using IBM SPSS 25.0. Continuous data of ASCO-VF scores were plotted and analyzed to assess the normality of the underlying distribution. Since ASCO-VF has no explicit definition for what score is deemed “meaningful value achieved”; we split scores at the 75th percentile of ASCO-VF scores as the cutoff score, referring to the meaningful value achieved of ESMO-MCBS as a grade of 4, 5, B, or A. We split the cutoff scores for subsequent analyses. A score above the cutoff was defined as “meaningful value achieved” while a score below the cutoff indicated “meaningful value not achieved”. Receiver operating characteristic (ROC) curves were generated to establish the predictive value of ASCO-VF score to meet ESMO-MCBS grade and discriminate the agreement of these two value assessment tools. Subgroups analyses were performed according to palliative and curative intent of the eligible trials. Spearman’s correlation was used to assess the association between monthly cost and ASCO-VF score/ESMO-MCBS grade. A *p* < 0.05 was deemed significant for all analyses.

## Results

### Eligible Studies and Characteristics

Of the 11,414 reports identified through search of electronic databases, 19 phase III RCTs eventually met our eligibility criteria (Borghaei et al., 2015; Brahmer et al., 2015; Motzer et al., 2015; Robert et al., 2015; Weber et al., 2015; Cella et al., 2016; Ferris et al., 2016; Herbst et al., 2016; Long et al., 2016; Reck et al., 2016; Bellmunt et al., 2017; Brahmer et al., 2017; Carbone et al., 2017; Harrington et al., 2017; Kang et al., 2017; Weber et al., 2017; Eggermont et al., 2018; Hellmann et al., 2018; Hodi et al., 2018; Reck et al., 2018; Reck et al., 2018; Shitara et al., 2018; Cohen et al., 2019; Mok et al., 2019; Wu et al., 2019). Of these, seven (36.8%) trials included treatments with nivolumab while 12 (63.2%) with pembrolizumab; eight (42.1%) trials involved treatments for non-small-cell lung cancer (NSCLC), five (26.3%) for melanoma, two (10.5%) for head and neck squamous cell carcinoma and gastric or gastro-esophageal junction cancer, respectively, and one each (5.3%) for urothelial cancer and renal-cell carcinoma, respectively. The longest follow-up time was 48 months of nivolumab for melanoma. The largest sample size was 1,537 of nivolumab for NSCLC (Table 1).

**TABLE 1:** Characteristics of included phase III randomized controlled trials of PD-1 inhibitor.

NO.	Year	Registry number	Study code	Disease type	Setting	Drug	PD-L1 expression level	Sample size	Follow-up time (m)	Outcomes	Industry sponsorship	ASCO-VF scores	ESMO-MCBS grade
1	2019	NCT02220894	KEYNOTE-042 (Cohen et al., 2019)	NSCLC	First-line for locally advanced or metastatic with EGFR and ALK WT	Pembrolizumab vs. paclitaxel or pemetrexed plus carboplatin	PD-L1≥1%	1,274 (637/637)	12.8	OS, PFS, ADEs, QALY	Yes	30.2	3
2	2019	NCT02252042	KEYNOTE-040 (Wu et al., 2019)	Head and neck squamous cell carcinoma	Second-line for recurrent or metastatic	Pembrolizumab vs. methotrexate, docetaxel, or cetuximab	——	495 (247/248)	7.5	ORR, OS, PFS, ADEs	Yes	30.8	3
3	2019	NCT02613507	CheckMate 078 (Shitara et al., 2018)	NSCLC	Second-line for platinum-based doublet chemo- therapywith EGFR and ALK WT	Nivolumab vs. docetaxel	——	504 (338/166)	8.8	ORR, OS, ADEs	Yes	47.8	3
4	2018	NCT02370498	KEYNOTE-061 (Eggermont et al., 2018)	Gastric or gastro-oesophageal junction cancer	Second-line for advanced gastric or gastro-oesophageal junction cancer	Pembrolizumab vs. Paclitaxel	PDL1 CPS≥1	395 (196/199)	8.5	ORR, OS, PFS, ADEs	Yes	30	3
5	2018	NCT02362594	EROTC1325/KEYNOTE-054 (Hodi et al., 2018)	Melanoma	Completely resected stage III	Pembrolizumab vs. placebo		1,019 (514/505)	15	RFS, OS, DFS, ADEs, QALY	Yes	9.4	A
6	2018	NCT01844505	CheckMate 067 (Hellmann et al., 2018)	Melanoma	First-line for stage III or IV with BRAF mutation	Nivolumab plus ipilimumab or nivolumab alone vs. Ipilimumab alone	——	945 (314/316/315)	48	ORR, OS, PFS, ADEs	Yes	38.8	5
7	2018	NCT02477826	CheckMate 227 (Bellmunt et al., 2017)	NSCLC	First-line for stage IV or recurrent with EGFR and ALK WT	Nivolumab plus ipilimumab, nivolumab monotherapy vs. platinum doublet chemotherapy	——	1,537 (576/391/570)	11.2	PFS, ADEs	Yes	36.3	2
8	2017	NCT02256436	KEYNOTE-045 (Carbone et al., 2017)	Urothelial cancer	Second-line for advanced	Pembrolizumab vs. paclitaxel, docetaxel, or vinflunine	——	542 (270/272)	14.1	ORR, OS, PFS, ADEs	Yes	40.9	5
9	2017	NCT02041533	CheckMate 026 (Weber et al., 2017)	NSCLC	First-line for stage IV or recurrent with EGFR and ALK WT	Nivolumab vs. platinum doublet chemotherapy	——	541 (270/271)	13.7	ORR, OS, PFS, ADEs	Yes	10.9	2
10	2017	NCT02388906	CheckMate 238 (Kang et al., 2017)	Melanoma	Adjuvant resected stage III or IV	Nivolumab vs. Ipilimumab	PD-L1≥5%	906 (453/453)	18	RFS, ADEs	Yes	29.6	A
11	2017	NCT02267343	ONO-4538–12, ATTRACTION-2 (Reck et al., 2016)	Gastric or gastro-oesophageal junction cancer	After second-line for advanced	Nivolumab vs. placebo	PD-L1≥1%	493 (330/163)	12	ORR, OS, PFS, ADEs	Yes	7	1
12	2016	NCT02142738	KEYNOTE-024 (Herbst et al., 2016; Brahmer et al., 2017)	NSCLC	First-line for stage IV with EGFR and ALK WT	Pembrolizumab vs. platinum-based chemotherapy	PD-L1≥50%	305 (154/151)	11.2	ORR, OS, PFS, ADEs, QALY	Yes	70	5
13	2016	NCT01905657	KEYNOTE-010 (Ferris et al., 2016)	NSCLC	Second-line after platinum-based therapy or TKIs(EGFR/ALK sensitive mutated)	Pembrolizumab vs. docetaxel	PD-L1≥1%	687 (344/343)	13.1	ORR, OS, PFS, ADEs, QALY	Yes	41.6	3
14	2016	NCT02105636	CheckMate 141 (Borghaei et al., 2015; Harrington et al., 2017)	Head and neck squamous-cell carcinoma	Platinum-refractory recurrent or metastatic	Nivolumab vs. methotrexate, docetaxel, cetuximab	PD-L1≥1%	381 (240/141)	16.8	ORR, OS, PFS, ADEs, QALY	Yes	82.5	4
15	2015	NCT01673867	CheckMate 057 (Brahmer et al., 2015; Reck et al., 2018)	Nonsquamous NSCLC	EGFR mutation/ALK translocation	Nivolumab vs. docetaxel	——	582 (292/290)	17.2	ORR, OS, PFS, ADEs, QALY	Yes	63.3	4
16	2015	NCT01642004	CheckMate 017 (Motzer et al., 2015; Reck et al., 2018)	Squamous-cell NSCLC	Second-line for stage IIIB or IV after platinum-based therapy	Nivolumab vs. docetaxel	——	272 (135/137)	11	ORR, OS, PFS, ADEs, QALY	Yes	78.5	5
17	2015	NCT01668784	CheckMate 025 (Weber et al., 2015; Cella et al., 2016)	Renal cell carcinoma	Advanced or metastatic	Nivolumab vs. everolimus	——	706 (362/344)	14	ORR, OS, PFS, ADEs, QALY	Yes	46.7	5
18	2015	NCT01721746	CheckMate 037 (Robert et al., 2015)	Melanoma	Second-line for unresectable stage IIIC or IV metastatic	Nivolumab vs. dacarbazine, or carboplatin plus paclitaxel	PD-L1≥5%	405 (272/133)	6	ORR, OS, PFS, ADEs	Yes	49.2	4
19	2014	NCT01721772	CheckMate 066 (Robert et al., 2015; Long et al., 2016)	Melanoma	Unresectable, previously untreated stage III or IV metastatic without BRAF mutation	Nivolumab vs. dacarbazine (double-blind)	PD-L1≥5%	418 (210/208)	73 weeks	ORR, OS, PFS, ADEs, QALY	Yes	94.7	4

*NSCLC: non-small-cell lung cancer; WT: wide type; RFS: recurrence-free survival; OS: overall survival; PFS: progression-free survival; ORR: Objective Response Rate; ADE: adverse events; EGFR: epidermal growth factor receptor; ALK: anaplastic lymphoma kinase.*

### Value Scores/Grades

**FIGURE 1:**
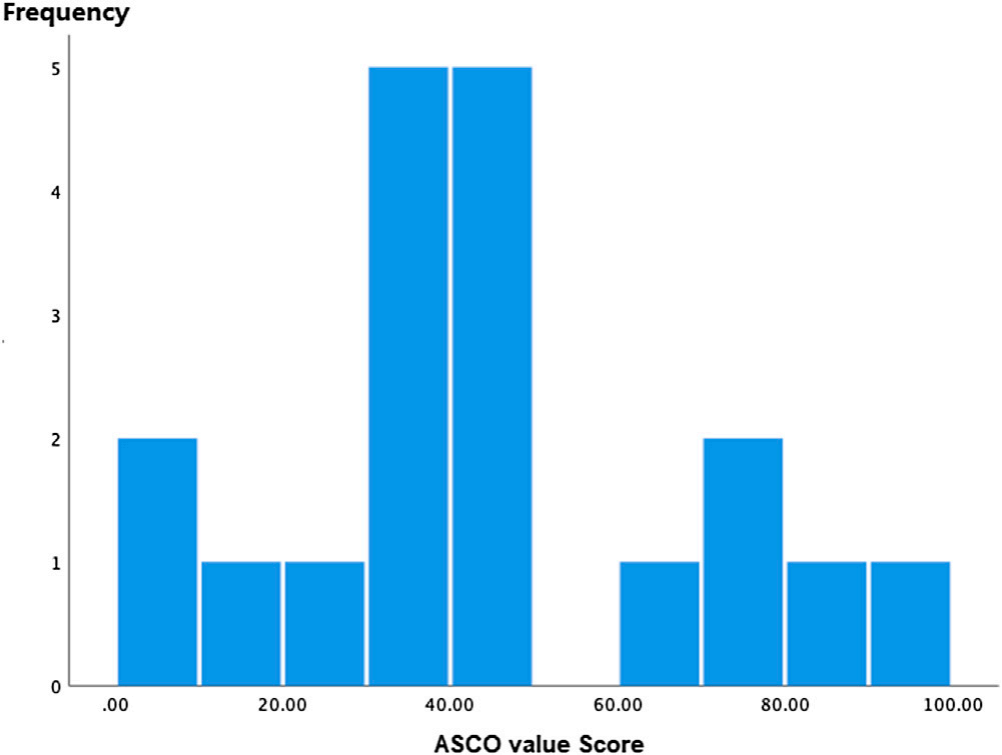
Distributions of the ASCO-VF Scores (Histograms).

ASCO-VF scores ranged from 7 to 94.7 (Figure 1), and the scores were normally distributed. Median ASCO-VF score was 40.90, with inter-quartile range (IQR) 33.30. Ten (52.6%) trials fell below, while nine (50.1%) trials were above. Since ASCO-VF has no explicit definition of what score is deemed “meaningful value achieved”; we split scores at the 75th percentile of ASCO-VF scores—63.3 as the cutoff score, referring to the meaningful value achieved of ESMO-MCBS as a grade of 4, 5, B, or A. Therefore, five (26.3%) trials were above the threshold whereas 14 (73.3%) fell below. Eleven (57.9%) of the 19 RCTs met the ESMO-MCBS criteria for meaningful value achieved. Of the 14 trials that did not meet the ASCO-VF cutoff score, only eight did not meet the ESMO-MCBS “meaningful value achieved” criteria.

### Association Between ASCO-VF and ESMO-MCBS

**FIGURE 2:**
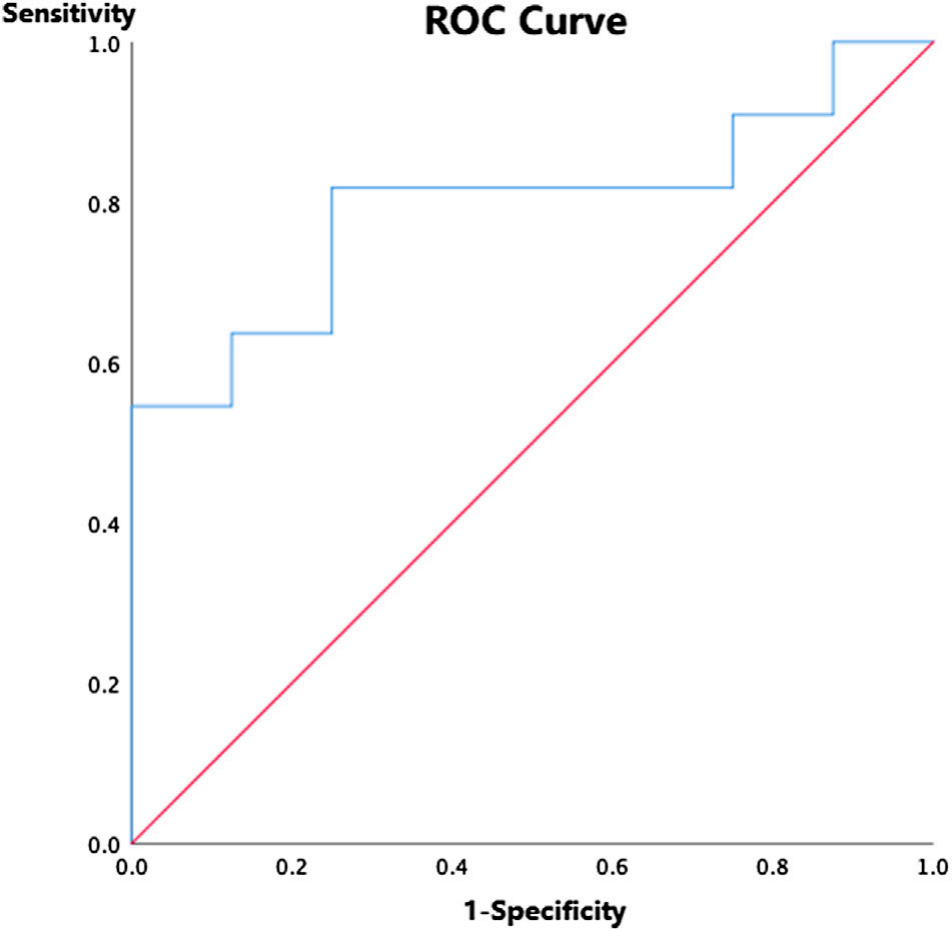
Receiver operating characteristic curve for ASCO-VF scores and ESMO-MCBS grades among 19 randomized controlled trials.

ROC curve was used to establish a discrimination threshold of ASCO-VF scores in relation to the ESMO-MCBS criteria, and the threshold score was approximately 40, which was comparatively close to the median ASCO-VF value scores. Nevertheless, the area under the curve was 0.795 (*p*＜0.05) (Figure 2), suggesting only fair predictive value. Agreement between ASCO-VF and ESMO-MCBS thresholds was only fair (*κ* = 0.412, *p*＜0.05).

### Correlation Between Cost and Value Results

Incremental monthly cost data, ranged from ¥28,426.40 to ¥47,661.88, were not normally distributed and were analyzed with non-parametric statistics. Incremental monthly cost and ASCO-VF scores were negatively correlated (Spearman’s *ρ*= −0.272; *p* = 0.260), and a negative correlation was also noted between incremental monthly cost and ESMO-MCBS grades (Spearman’s *ρ*= −0.088; *p* = 0.720).

## Discussion

### Summary of Key Findings

To the best of our knowledge, this was the first study that applied ASCO-VF and ESMO-MCBS to comprehensively address the value of two PD-1 inhibitors, nivolumab and pembrolizumab, and offer insight into the association between these two frameworks.

Most studies focused on NSCLS and melanoma. In addition to NSCLC, melanoma, head and neck squamous cell carcinoma and gastric or gastro-esophageal junction cancer, only one trial compared nivolumab in renal cell carcinoma while another compared pembrolizumab in urothelial cancer (Cella et al., 2016).

There were some conflicting results. For gastric or gastro-esophageal junction cancer, ASCO-VF scores and ESMO-MCBS grades showed that both nivolumab and pembrolizumab were of little value, with ASCO-VF scores 7 vs. ESMO-MCBS grade 1 for nivolumab (Reck et al., 2016) and ASCO-VF scores 30 vs. ESMO-MCBS grade 3 for pembrolizumab (Eggermont et al., 2018). For the second-line treatment for recurrent or metastatic head and neck squamous cell carcinoma, nivolumab (ASCO-VF scores 82.5 vs. ESMO-MCBS grade 4) was valuable (Borghaei et al., 2015; Harrington et al., 2017), but pembrolizumab was not (ASCO-VF scores 30.8 vs. ESMO-MCBS grade 3) (Wu et al., 2019). For melanoma, discordant ASCO-VF scores and ESMO-MCBS grades were generated except CheckMate 066 (Robert et al., 2015; Long et al., 2016) and CheckMate 037 (Robert et al., 2015) examining the efficacy and safety nivolumab for melanoma. For CheckMate 066, ASCO-VF score was highest and ESMO-MCBS grade was only 4. For NSCLC, results were almost the same with ASCO-VF scores and ESMO-MCBS grades. For CheckMate 017 (Motzer et al., 2015; Reck et al., 2018) examining the second-line treatment of nivolumab for squamous-cell stage IIIB or IV NSCLC after platinum-based therapy, and KEYNOTE-024 (Herbst et al., 2016; Brahmer et al., 2017) examining the first-line pembrolizumab treatment for stage IV NSCLC with EGFR and ALK wild type, both ASCO-VF scores and ESMO-MCBS grades were high, demonstrating that they were valuable.

### Strengths and Limitations

This study fills a crucial knowledge gap regarding the value of PD-1 inhibitors among cancer patients. Several strengths should be noted. Firstly, each trial had a wide range of ASCO-VF scores. Secondly, not all trials met the ESMO-MCBS “meaningful value achieved” criteria. Thirdly, only a fair association was found between ASCO-VF and ESMO-MCBS. Fourth, there was no correlation between incremental monthly cost and ASCO-VF scores or ESMO-MCBS grades.

Irrespective of whether the trials described patients with specific PD-1 expression level, high ASCO-VF scores and ESMO-MCBS grades were recorded.

Nevertheless, this study also had several limitations. Firstly, the ASCO-VF score and ESMO-MCBS grade of the trials with the largest sample size was not high and did not meet the ASCO-VF cutoff score and the ESMO-MCBS “meaningful value achieved” criteria. Secondly, the association between ASCO-VF and ESMO-MCBS was only moderate in a cohort of 19 trials, substantially similar to those reported by others except one study including only a small number of trials (*n* = 5). Extensive efforts are needed to improve convergence of the two value assessment tools based on our findings. Thirdly, since ASCO-VF has no explicit definition of what score is deemed “meaningful value achieved”; we split scores at the 75th percentile of ASCO-VF scores—63.3 as the cutoff score, referring to the meaningful value achieved of ESMO-MCBS as a grade of 4, 5, B, and A. Nevertheless, changing this cutoff score will change the degree of correlation between these two tools.

### Clinical and Research Implications

This study sheds light on the important clinical issue about the comparative value of PD-1-related treatment. Our results demonstrated the value of a drug should not be judged solely by its price. It is necessary to conduct value assessment to insight the date beyond RCTs.

## Conclusion

There is only fair correlation between ASCO-VF and ESMO-MCBS. Not all treatments with nivolumab and pembrolizumab meet valuable thresholds according to value assessment tools established by ASCO or ESMO. Given the high prices of these drugs, valuable, appropriate and affordable treatment is important for decision-making.

## Data Availability Statement

The original contributions presented in the study are included in the article/Supplementary Material, further inquiries can be directed to the corresponding author.

## Author Contributions

QJ study design, drafting out and revising the manuscript critically for important intellectual content. MF study design, analysis and method directing. YPL participated in its design, revising the manuscript. JYL analysis and interpretation of data. HW analysis and interpretation of data. QJ and TY: conceiving of the study, revising the manuscript ﬁnally.

## Funding

Beijing Medical and Health Foundation (B20021CS).

## Conflict of Interest

The authors declare that the research was conducted in the absence of any commercial or financial relationships that could be construed as a potential conflict of interest.
